# Supplementary material for: Alkaline-SDS cell lysis of microbes with acetone protein precipitation for proteomic sample preparation in 96-well plate format
Source: PLoS One. 2023 Jul 7;18(7):e0288102. doi: 10.1371/journal.pone.0288102 (PMC10328223; doi:10.1371/journal.pone.0288102)
Supplement: S1 File — Also available on protocols.io. (PDF) [file pone.0288102.s001.pdf]

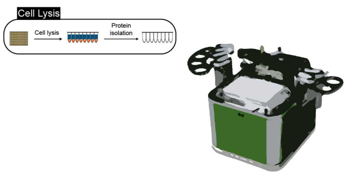

Alkaline-SDS cell lysis of microbes with acetone protein precipitation for proteomic sample preparation in 96-well plate format V.1

Forked from Acetone-Salt Protein Extraction for bacteria and fungi - 1.7mL tube

Yan Chen[1], Jennifer Gin[1], Christopher J Petzold[1]

[1]Lawrence Berkeley National Laboratory

LBNL omics   Agile BioFoundry   1 more workspace

Jennifer Gin
Lawrence Berkeley National Laboratory

VERSION 1

JAN 18, 2023

OPEN ACCESS

DOI: dx.doi.org/10.17504/protocols.io.6qpvr6xjpvmk/v1

Protocol Citation: Yan Chen, Jennifer Gin, Christopher J Petzold 2023. Alkaline-SDS cell lysis of microbes with acetone protein precipitation for proteomic sample preparation in 96-well plate format. **protocols.io** https://dx.doi.org/10.17504/protocols.io.6qpvr6xjpvmk/v1

License: This is an open access protocol distributed under the terms of the Creative Commons Attribution License, which permits unrestricted use, distribution, and reproduction in any medium, provided the original author and source are credited

Protocol status: Working
We use this protocol and it's working

Created: Dec 09, 2021

Last Modified: Jan 18, 2023

PROTOCOL integer ID: 55810

ABSTRACT

This high-throughput protocol details the steps to extract protein from Gram-negative bacteria, Gram-positive bacteria, or non-filamentous fungi in 96-well plate format for quantitative proteomic workflows. This protocol uses a bench-top automated liquid dispenser but the volumes and times also apply to manual and multi-channel pipetter use. This protocol is designed for lab-based, culture conditions and synthetic community experiments where complex sample matrices are minimized. Additional sample preservation and/or protein extraction methods may be required for environmental samples (e.g., feces, soil) to minimize protein degradation and maintain sample integrity.

This protocol works best as part of a high-throughput proteomic sample preparation workflow with:

Automated Protein Quantitation with the Biomek-FX liquid handler system

and

Automated Protein Normalization and Tryptic Digestion on a Biomek-NX Liquid Handler System

Keywords: Proteomics, Sample preparation, Bacteria, Fungi, Protein extraction

GUIDELINES

- All centrifuge steps use an Eppendorf 5810R centrifuge.
- You can use Qiagen Lysis Buffer P2 (Qiagen, Cat. #19052) as the alkaline-SDS cell lysis buffer.

Note

Increase reagent volumes accordingly when processing larger amounts of biomass. This protocol works in larger formats (e.g., 1.7 mL Eppendorf tubes) too.

MATERIALS

PCR Plate 96-well non-skirted Thermo Fisher Scientific Catalog #AB0600

Hydrochloric acid Sigma Catalog #320331
Sodium Hydroxide (200 mM)
1% Sodium Dodecyl Sulfate (SDS)
Ammonium bicarbonate LC-MS grade VWR Scientific Catalog #BJ40867-50G
Benzonase nuclease Millipore Catalog #70746
Acetone Sigma Catalog #179124
LC-MS grade Methanol VWR Scientific, Catalog #BJLC230-2.5

Optional:
- Qiagen Lysis Buffer P2 (Qiagen, Cat.#19052) in place of NaOH and SDS

SAFETY WARNINGS

Acetone is used in this protocol so please follow the appropriate safety guidelines for handling and disposing of non-halogenated solvents at your institution.

Sodium Hydroxide is a HIGHLY CORROSIVE CHEMICAL and contact can severely irritate and burn the skin and eyes with possible eye damage. Inhaling Sodium Hydroxide can irritate the lungs.

Wear gloves and appropriate PPE for safety and to minimize contamination of samples.

BEFORE START INSTRUCTIONS

For this protocol you will need:
- a bench-top automated liquid dispenser (*e.g.*, Formulatrix Mantis) or manual/multi-channel pipetters
- an Eppendorf 5810R centrifuge with S-4-104 rotor or similar centrifuge

Mix at least 3 mL of NaOH/SDS buffer for final concentrations of:
- 200 mM NaOH
- 1% SDS
or use Qiagen Lysis Buffer P2 (Qiagen, Cat.#19052)

Cell lysis

6m

1 Start with 10 µL of cells per well a non-skirted PCR plate (Thermo Scientific, Cat.#AB0600).

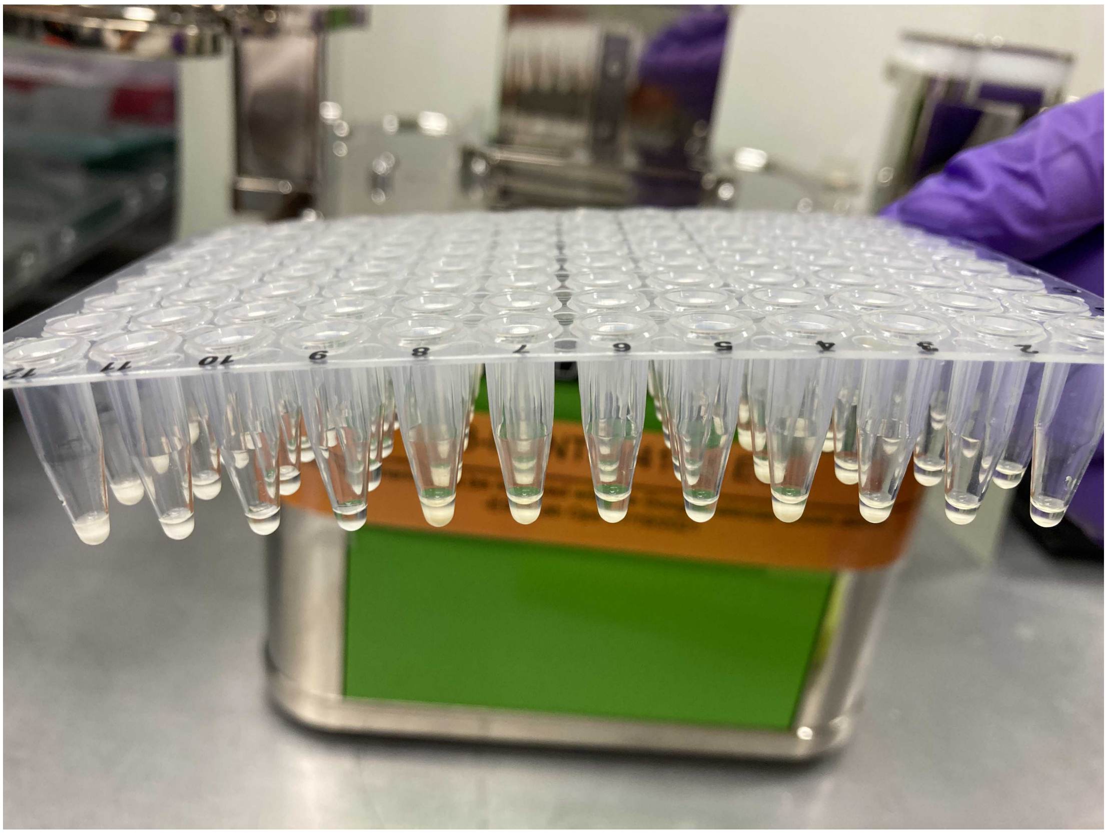

Plate of cell pellets

2 Add 25 µL of alkaline-SDS cell lysis buffer (200 mM NaOH, 1% SDS) to each well. 1m

3 Resuspend the cell pellet in lysis buffer on a plate mixer. 5m

Note

Ensure proper mixing of the cell lysis buffer and the cell pellet.

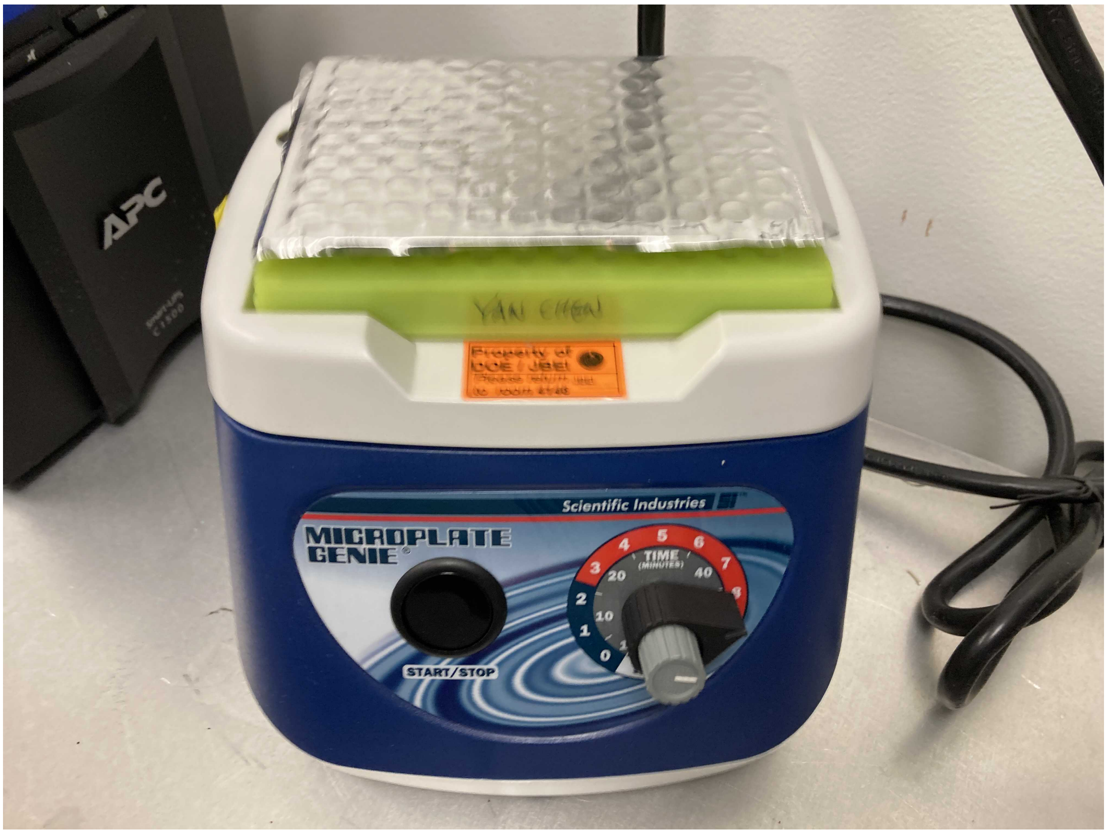
APC
YAN CHEN
Scientific Industries
MICROPLATE GENIE
TIME (MINUTES)
START/STOP

Plate mixer

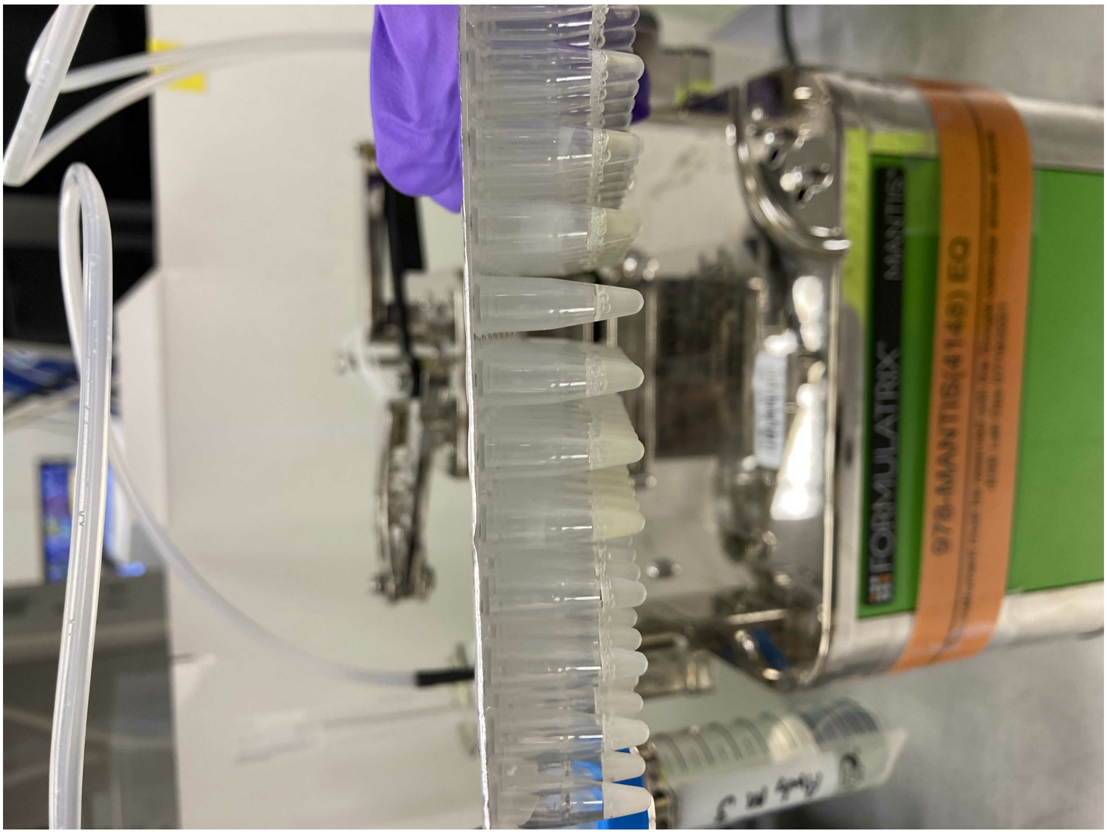

Cells resuspended in lysis buffer

Neutralization and Benzonase treatment

8m

4 Add 2.75 µL [M] 1 Molarity (M) Hydrochloric acid (Sigma Cat.#320331) to each well. 2m

5 Add 25 µL 100 mM Ammonium bicarbonate (VWR Scientific Cat.#BJ40867-50G) and 0.5 µL Benzonase nuclease (Millipore Cat.#70746) to each well. 3m

6 Mix thoroughly on the plate mixer. 3m

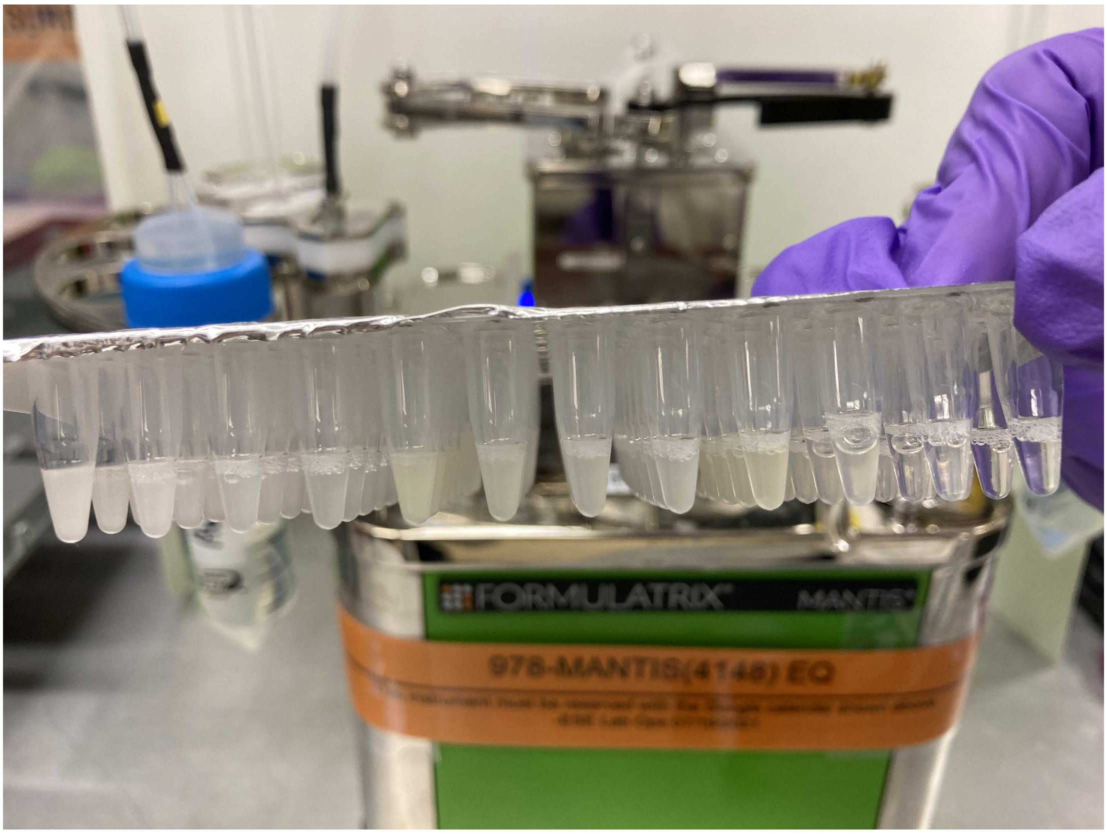

Cell lysate after benzonase treatment

Salt-Acetone protein precipitation

8m

7 Add 200 µL 100% Acetone (Sigma Cat.#179124) to each well and let sit at room temperature for 5 minutes.

5m

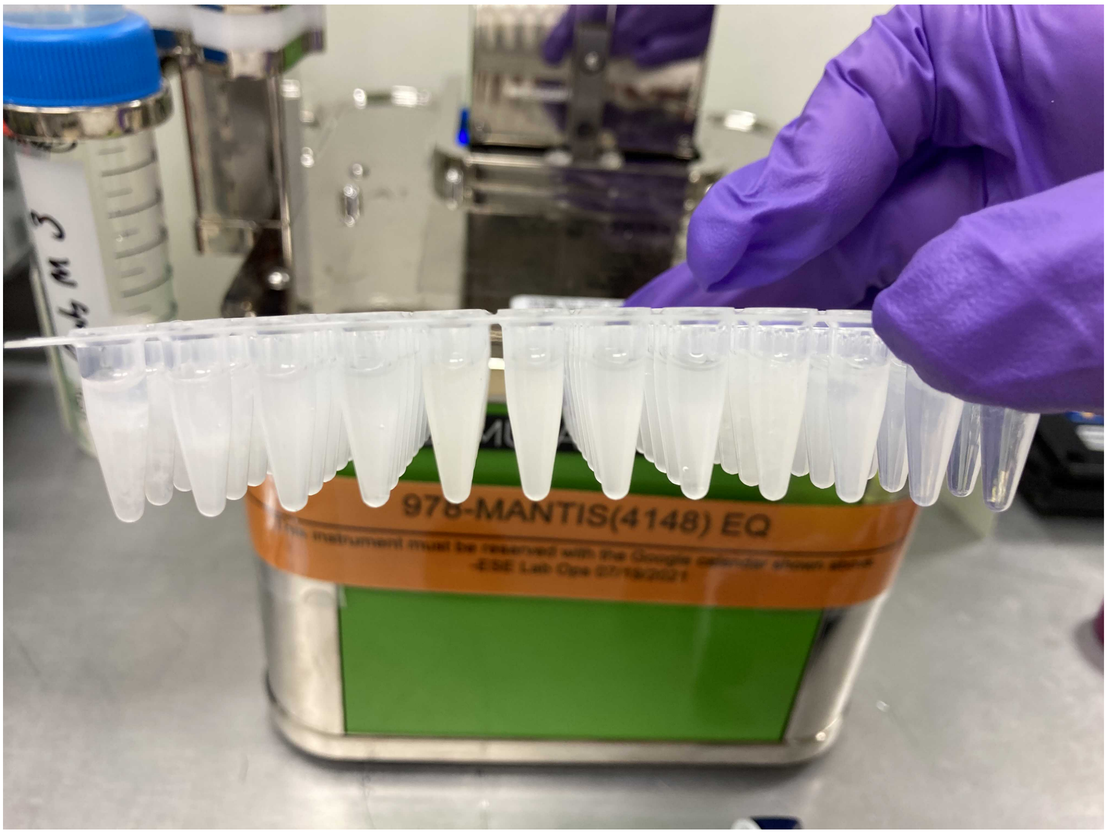

Protein precipitation after mixing in 100% acetone

8 Centrifuge at 4000 rpm, 00:02:00 .

2m

9 Remove supernatant.

1m

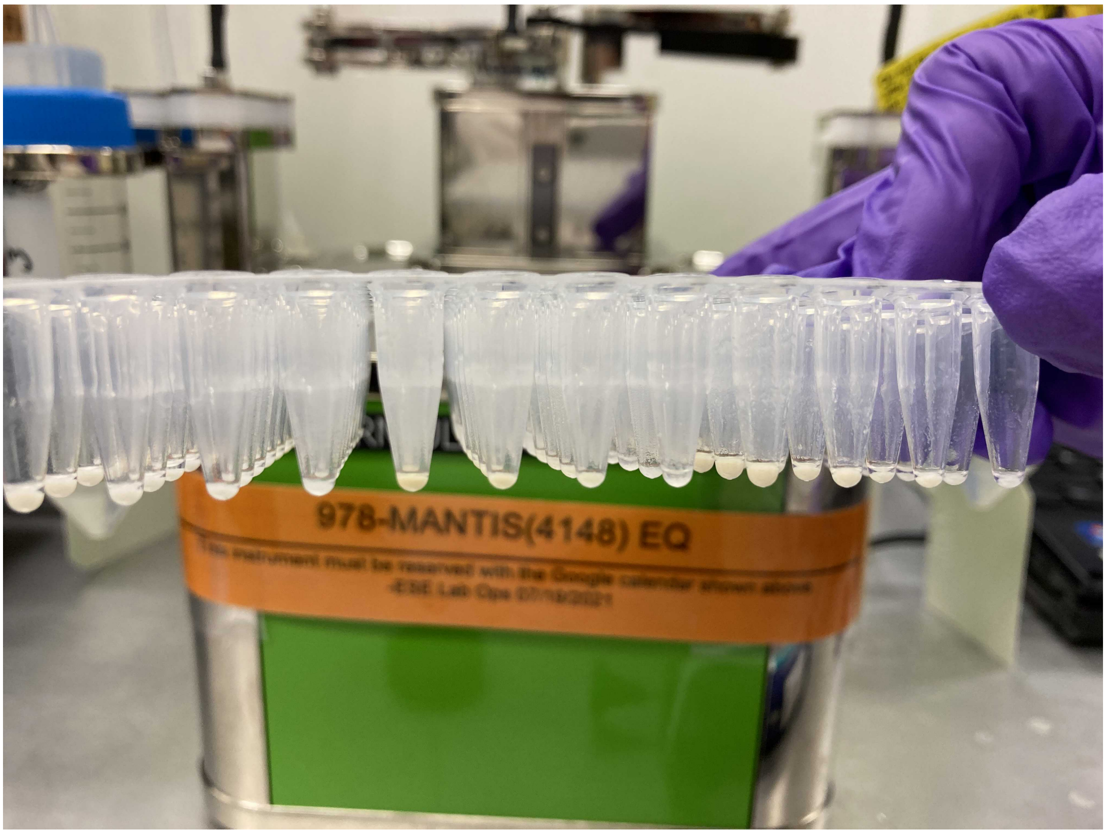

Protein pellet after centrifugation and removal of supernatant

Wash and resuspend protein

8m

10 Wash protein pellet twice using 150 µL 80% Acetone (Sigma Cat.#179124) . 6m

Note

Increase the number of washes depending on the sample matrix background.

11 Add 60 µL of [M] 100 millimolar (mM) Ammonium bicarbonate in 10% Methanol to each well to resuspend protein pellet. 2m

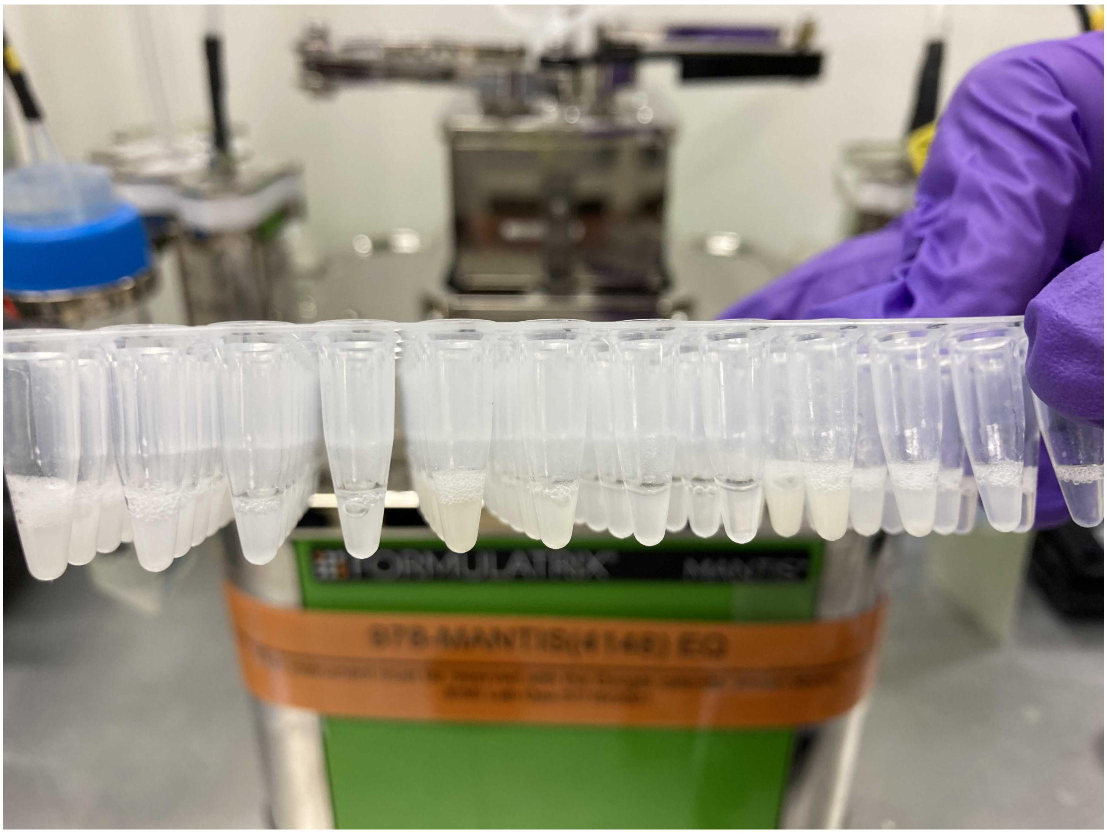

Protein pellet resuspended in 60 uL of Ammonium bicarbonate in 10% Methanol

12 Store at -20 °C until ready for [Automated Protein Quantitation with the Biomek-FX liquid handler system](https://dx.doi.org/10.17504/protocols.io.6qpvr6xjpvmk/v1).
