# Supplementary material for: Alkaline-SDS cell lysis of microbes with acetone protein precipitation for proteomic sample preparation in 96-well plate format
Source: PLoS One. 2023 Jul 7;18(7):e0288102. doi: 10.1371/journal.pone.0288102 (PMC10328223; doi:10.1371/journal.pone.0288102)
Supplement: Attachment — Submitted filename: PONE-D-23-03070_reviewer comments.pdf [file pone.0288102.s002.pdf]

Manuscript Number PONE-D-23-03070

Alkaline-SDS cell lysis of microbes with acetone protein precipitation for proteomic sample preparation in 96-well plate format

Recommendation: Publish after resubmission.

Comments:

Abstract: Authors should be refurnished abstract.

1. Authors should be checked grammar “in alkaline chemical buffer” or “in an alkaline chemical buffer”?

Introduction: Authors should be rechecked

Result sections:

1. You have extracted protein from Gram-positive bacteria species, Gram-negative bacteria species, and non-filamentous fungi species with ~2x109 cells of biomass from each species but why did Gram-positive bacteria species total protein obtained very less than Gram-negative species and non-filamentous?. Does protein extraction concentration depend on species? Any specification on the extraction?

2. Haven’t tested any animal cell line/tissues. You should test on primary cells or cell lines because that is good for justification for microbial protocol. The author should at least do the test on either animal tissues/primary cells/cell lines. It may be not applicable to animal cells.

Figure Captions:

1. Fig.1: Error bars should be represented by a minimum of three replicates performed on a separate day. Any justification for why the authors' error bars represented two replicates performed/experiments?
