# Supplementary material for: Alkaline-SDS cell lysis of microbes with acetone protein precipitation for proteomic sample preparation in 96-well plate format
Source: PLoS One. 2023 Jul 7;18(7):e0288102. doi: 10.1371/journal.pone.0288102 (PMC10328223; doi:10.1371/journal.pone.0288102)
Supplement: Attachment — Submitted filename: PONE-D-23-03070_reviewed.pdf [file pone.0288102.s003.pdf]

PLOS ONE

Alkaline-SDS cell lysis of microbes with acetone protein precipitation for proteomic sample preparation in 96-well plate format

--Manuscript Draft--

**Manuscript Number:**	PONE-D-23-03070
**Article Type:**	Lab Protocol
**Full Title:**	Alkaline-SDS cell lysis of microbes with acetone protein precipitation for proteomic sample preparation in 96-well plate format
**Short Title:**	Alkaline-SDS cell lysis of microbes with acetone protein precipitation in 96-well plate format
**Corresponding Author:**	Christopher J. Petzold<br>Lawrence Berkeley Laboratory: E O Lawrence Berkeley National Laboratory<br>Emeryville, CA UNITED STATES
**Keywords:**	Sample preparation; Proteomics; Automation; Bacteria; Fungi; Microbes; Biotechnology; Synthetic microbial communities; High-throughput
**Abstract:**	Plate-based proteomic sample preparation offers a solution to the large sample throughput demands in the biotechnology field where hundreds or thousands of engineered microbes are constructed for testing is routine. Meanwhile, sample preparation methods that work efficiently on broader microbial groups are desirable for new applications of proteomics in other fields, such as microbial communities. Here, we detail a step-by-step protocol that consists of cell lysis in alkaline chemical buffer (NaOH/SDS) followed by protein precipitation with high-ionic strength acetone in 96-well format. The protocol works for a broad range of microbes (e.g., Gram-negative bacteria, Gram-positive bacteria, non-filamentous fungi) and the resulting proteins are ready for tryptic digestion for bottom-up quantitative proteomic analysis without the need for desalting column cleanup. The yield of protein using this protocol increases linearly with respect to the amount of starting biomass from 0.5 - 2.0 OD*mL of cells. By using a bench-top automated liquid dispenser, a cost-effective and environmentally-friendly option to eliminating pipette tips and reducing reagent waste, the protocol takes approximately 30 minutes to extract protein from 96 samples. Tests on mock mixtures showed expected results that the biomass composition structure is in close agreement with the experimental design. Lastly, we applied the protocol for the composition analysis of a synthetic community of environmental isolates grown on two different media. This protocol has been developed to facilitate rapid, low-variance sample preparation of hundreds of samples and allow flexibility for future protocol development.
**Order of Authors:**	Yan Chen
	Jennifer W. Gin
	Ying Wang
	Markus de Raad
	Stephen Tan
	Nathan J. Hillson
	Trent R. Northen
	Paul D. Adams
	Christopher J. Petzold
**Additional Information:**	
**Question**	**Response**
**Financial Disclosure**<br><br>Enter a financial disclosure statement that	The funders had and will not have a role in study design, data collection and analysis, decision to publish, or preparation of the manuscript. The United States Government retains and the publisher, by accepting the article for publication, acknowledges that

describes the sources of funding for the work included in this submission. Review the submission guidelines for detailed requirements. View published research articles from *PLOS ONE* for specific examples.<br><br>This statement is required for submission and **will appear in the published article** if the submission is accepted. Please make sure it is accurate.<br><br>**Unfunded studies**<br>Enter: *The author(s) received no specific funding for this work.*<br><br>**Funded studies**<br>Enter a statement with the following details:<br>• Initials of the authors who received each award<br>• Grant numbers awarded to each author<br>• The full name of each funder<br>• URL of each funder website<br>• Have or will the sponsors or funders play any role in the study design, data collection (when applicable) and analysis, decision to publish, or preparation of the manuscript?<br>• **NO** - Include this sentence at the end of your statement: *The funders had and will not have a role in study design, data collection and analysis, decision to publish, or preparation of the manuscript.*<br>• **YES** - Specify the role(s) played.<br><br>* typeset	the United States Government retains a non-exclusive, paid-up, irrevocable, worldwide license to publish or reproduce the published form of this manuscript, or allow others to do so, for United States Government purposes. The views and opinions of the authors expressed herein do not necessarily state or reflect those of the United States Government or any agency thereof. Neither the United States Government nor any agency thereof, nor any of their employees, makes any warranty, expressed or implied, or assumes any legal liability or responsibility for the accuracy, completeness, or usefulness of any information, apparatus, product, or process disclosed, or represents that its use would not infringe privately owned rights. The proof-of-concept work with mono-cultures and resources were part of the Joint BioEnergy Institute (JBEI; http://www.jbei.org), the extension of the procedure and identification of the sources of error were part of the Agile BioFoundry (ABF; http://agilebiofoundry.org), and the application to synthetic microbial communities was part of Ecosystems and Networks Integrated with Genes and Molecular Assemblies (ENIGMA; http://enigma.lbl.gov), a Science Focus Area led by Lawrence Berkeley National Laboratory. This work was supported by the U.S. Department of Energy, Office of Science, Office of Biological & Environmental Research (JBEI and ENIGMA) and through the Bioenergy Technology Office within the DOE Office of Energy Efficiency and Renewable Energy (ABF) through contract DE-AC02-05CH11231 between Lawrence Berkeley National Laboratory and the U. S. Department of Energy.
**Competing Interests**<br><br>Use the instructions below to enter a competing interest statement for this submission. On behalf of all authors, disclose any competing interests that could be perceived to bias this work—acknowledging all financial support and any other relevant financial or non-financial competing interests.<br><br>This statement is required for submission and **will appear in the published article** if the submission is accepted. Please make sure it is accurate and that any funding	N.J.H. has financial interests in TeselaGen Biotechnologies, and Ansa Biotechnologies. The other authors have declared that no competing interests exist.

sources listed in your Funding Information later in the submission form are also declared in your Financial Disclosure statement.<br><br>View published research articles from *PLOS ONE* for specific examples.<br><br>**NO authors have competing interests**<br><br>Enter: *The authors have declared that no competing interests exist.*<br><br>**Authors with competing interests**<br><br>Enter competing interest details beginning with this statement:<br><br>*I have read the journal's policy and the authors of this manuscript have the following competing interests: [insert competing interests here]*<br><br>* typeset	
**Ethics Statement**<br><br>Enter an ethics statement for this submission. This statement is required if the study involved:<br><br>• Human participants<br>• Human specimens or tissue<br>• Vertebrate animals or cephalopods<br>• Vertebrate embryos or tissues<br>• Field research<br><br>Write "N/A" if the submission does not require an ethics statement.<br><br>General guidance is provided below. Consult the submission guidelines for detailed instructions. **Make sure that all information entered here is included in the Methods section of the manuscript.**	N/A

Powered by Editorial Manager® and ProduXion Manager® from Aries Systems Corporation

**Format for specific study types**<br><br>**Human Subject Research (involving human participants and/or tissue)**<br>• Give the name of the institutional review board or ethics committee that approved the study<br>• Include the approval number and/or a statement indicating approval of this research<br>• Indicate the form of consent obtained (written/oral) or the reason that consent was not obtained (e.g. the data were analyzed anonymously)<br><br>**Animal Research (involving vertebrate animals, embryos or tissues)**<br>• Provide the name of the Institutional Animal Care and Use Committee (IACUC) or other relevant ethics board that reviewed the study protocol, and indicate whether they approved this research or granted a formal waiver of ethical approval<br>• Include an approval number if one was obtained<br>• If the study involved *non-human primates*, add *additional details* about animal welfare and steps taken to ameliorate suffering<br>• If anesthesia, euthanasia, or any kind of animal sacrifice is part of the study, include briefly which substances and/or methods were applied<br><br>**Field Research**<br><br>Include the following details if this study involves the collection of plant, animal, or other materials from a natural setting:<br>• Field permit number<br>• Name of the institution or relevant body that granted permission	
**Data Availability**<br><br>Authors are required to make the data underlying their research findings fully available, without restriction.<br><br>If the manuscript reports sample data generated using the protocol, the data underlying those results must be made available at the time of publication. A Data	Yes - sample data are fully available without restriction

Powered by Editorial Manager® and ProduXion Manager® from Aries Systems Corporation

Availability Statement describing where the data can be found is required at submission. When legal or ethical requirements prohibit public data sharing, authors must indicate how researchers may obtain access to the data. See the PLOS Data Policy and FAQ for detailed information.<br><br>A Data Availability Statement describing where the data can be found is required at submission. Your answers to this question constitute the Data Availability Statement and **will be published in the article**, if accepted.<br><br>**Important:** Stating 'data available on request from the author' is not sufficient. If your data are only available upon request, select 'No' for the first question and provide justification in the text box.<br><br>For protocols without sample data or results, you must state this in your Data Availability Statement.<br><br>Do the authors confirm that EITHER the protocol does not report results OR that sample data will be made available?	
**Describe where the data may be found in full sentences. If you are copying our sample text, replace any instances of XXX with the appropriate details.**<br><br>• If the data are **held or will be held in a public repository**, include URLs, accession numbers or DOIs. If this information will only be available after acceptance, indicate this by ticking the box below. For example: *All XXX files are available from the XXX database (accession number(s) XXX, XXX.)*.<br>• If the data are all contained **within the manuscript and/or Supporting Information files**, enter the following: *All relevant data are within the*	The LCMS analysis raw data have been deposited to the ProteomeXchange Consortium data depository at http://www.proteomexchange.org/. They are publicly accessible with the dataset identifier PXD039268.

manuscript and its Supporting Information files.<br>• If neither of these applies but you are able to provide **details of access elsewhere**, with or without limitations, please do so. For example:<br><br>*Data cannot be shared publicly because of [XXX]. Data are available from the XXX Institutional Data Access / Ethics Committee (contact via XXX) for researchers who meet the criteria for access to confidential data.*<br><br>*The data underlying the results presented in the study are available from (include the name of the third party and contact information or URL).*<br>• This text is appropriate if the data are owned by a third party and authors do not have permission to share the data.<br><br>* typeset	
Additional data availability information:	Tick here if the URLs/accession numbers/DOIs will be available only after acceptance of the manuscript for publication so that we can ensure their inclusion before publication.

Powered by Editorial Manager® and ProduXion Manager® from Aries Systems Corporation

Manuscript

Click here to access/download;Manuscript;Manuscript-Alkaline-SDS_cell_lysis_protocol-20230203.docx

Alkaline-SDS cell lysis of microbes with acetone protein precipitation for proteomic sample preparation in 96-well plate format

Yan Chen,[1,2,3] Jennifer W. Gin,[1,2,3] Ying Wang,[4] Markus de Raad,[4] Stephen Tan,[1,2,3] Nathan J. Hillson,[1,2,3] Trent R. Northen,[2,4] Paul D. Adams,[2,5,6] and Christopher J. Petzold[1,2,3,]*

[1]Biological Systems and Engineering Division, Lawrence Berkeley National Laboratory, Berkeley, CA, USA
[2]DOE Joint BioEnergy Institute, Emeryville, CA, USA
[3]DOE Agile BioFoundry, Emeryville, CA, USA
[4]Environmental Genomics and Systems Biology Division, Lawrence Berkeley National Laboratory, Berkeley, CA, USA
[5]Department of Bioengineering, University of California Berkeley, Berkeley, CA, USA
[6]Molecular Biophysics and Bioimaging Division, Lawrence Berkeley National Laboratory, Berkeley, CA, USA

KEYWORDS: Sample preparation, Proteomics, Automation, Bacteria, Fungi, Microbes, Biotechnology, Synthetic microbial communities, High-throughput

Abstract

Plate-based proteomic sample preparation offers a solution to the large sample throughput demands in the biotechnology field where hundreds or thousands of engineered microbes are constructed for testing is routine. Meanwhile, sample preparation methods that work efficiently on broader microbial groups are desirable for new applications of proteomics in other fields, such as microbial communities. Here, we detail a step-by-step protocol that consists of cell lysis in alkaline chemical buffer (NaOH/SDS) followed by protein precipitation with high-ionic strength acetone in 96-well format. The protocol works for a broad range of microbes (*e.g.*, Gram-negative bacteria, Gram-positive bacteria, non-filamentous fungi) and the resulting proteins are ready for tryptic digestion for bottom-up quantitative proteomic analysis without the need for desalting column cleanup. The yield of protein using this protocol increases linearly with respect to the amount of starting biomass from 0.5 - 2.0 OD*mL of cells. By using a bench-top automated liquid

dispenser, a cost-effective and environmentally-friendly option to eliminating pipette tips and reducing reagent waste, the protocol takes approximately 30 minutes to extract protein from 96 samples. Tests on mock mixtures showed expected results that the biomass composition structure is in close agreement with the experimental design. Lastly, we applied the protocol for the composition analysis of a synthetic community of environmental isolates grown on two different media. This protocol has been developed to facilitate rapid, low-variance sample preparation of hundreds of samples and allow flexibility for future protocol development.

Introduction

As new applications of protein analysis increase there is a concurrent impact on analytics, such as bottom-up, quantitative proteomics, to assay the samples. The impact of this is felt most intensely in biotechnology where the construction of hundreds or thousands of samples for testing is routine [1] and for analyses where the structure of microbial communities of diverse organisms must be assayed [2]. Consequently, it is crucial to improve the quality and throughput of sample preparation methods that facilitate such assays. Recently, multiple groups have automated various steps (*e.g.*, cell lysis, protein precipitation/isolation, protein quantification, tryptic digestion) of the common bottom-up proteomic sample preparation methods for various types of samples. There are a variety of automated protocols for the tryptic digestion and peptide cleanup steps [3–7] because once the protein is purified from the cells or matrices the digestion and cleanup steps are quite similar regardless of the original source.

In contrast, there are fewer examples of automation protocols for cell lysis or protein precipitation/isolation because the source of the protein sample greatly impacts those methods and commonly-used mechanical lysis techniques (*e.g.*, sonication, bead beating, French press) are challenging to automate. But, chemical lysis methods by using detergents or surfactants (*e.g.*, SDS, Triton) [8–11], organic solvents [12,13], acidification [14], chelating agents (EDTA), and chaotropic agents (*e.g.*, urea, guanidine) are more

amenable to automation. Recently, automated methods for FASP [8,15,16], SP3 [9], and several commercial options (*e.g.*, iST, S-Trap tips) leveraging these methods have been developed in plate format. These types of protocols effectively reduce both the total and hands-on time for sample preparation [11] but can become quite costly for large numbers of samples. Alternatively, cell lysis by using detergents under alkaline conditions coupled with purification by protein precipitation are rapid, easily automated, and cost effective. Work by Doucette and co-workers detail how high-yield (~98%) protein precipitation can be achieved in only two minutes by using a high ionic strength 80% acetone solution [17–19]. Here, we describe a rapid protocol for samples in 96-well plate format including cell lysis, benzonase digestion of the nucleic acids, and precipitation of the proteins with the salt-acetone method [17] that is applicable to monocultures and microbial communities. Even though SDS is used for lysis, protein precipitation and subsequent washing steps efficiently remove the SDS from the samples, consequently, clean-up steps are not necessary prior to LC-MS analysis. After resuspension of the proteins, we quantify the amount of protein extracted and show a linear increase of protein released from *Escherichia coli*, *Pseudomonas putida*, *Streptomyces albus*, *Corynebacterium glutamicum*, *Saccharomyces cerevisiae*, and *Rhodosporidium toruloides*. Next, we used a previously described automation protocol [20] to normalize the amount of protein and set up tryptic digestion and then analyzed the peptides with a short 10-minute gradient LC-MS/MS data independent acquisition (DIA) method [21] for both single organisms and mixtures of organisms. Lastly, we analyzed a synthetic community of five bacteria (four Gram-negative, one Gram-positive) isolated from the environment [22] to test the methods for composition analysis by quantifying the contribution of each of the species to the total biomass. This protocol, combined with previously established automated protein quantification and protein normalization protocols, provides a rapid, cost-effective method to prepare LC-MS proteomic samples from bacteria and non-filamentous fungi cell cultures.

Materials and Methods

The protocol described in this peer-reviewed article is published on protocols.io, https://www.protocols.io/view/alkaline-sds-cell-lysis-of-microbes-with-acetone-p-6qpvr6xjpvmk/v1 and is included for printing as Supporting Information File 1 (S1) with this article.

Expected results

The alkaline-SDS cell lysis with acetone protein precipitation protocol (S1 File) is composed of cell lysis with a mixture of 200 mM NaOH and 1% sodium dodecyl sulfate (SDS), neutralization with HCl, benzonase treatment to remove nucleic acids, followed by the addition of acetone (80% by volume) to precipitate the proteins. The protocol takes approximately 30 minutes to process one full 96-well plate of samples, including centrifugation steps. The protocol time is scalable to the number of samples to be processed. Application of this protocol with an automated liquid dispenser offers cost-savings by reducing the number of pipette tips used in the protocol and minimizing reagent waste due to extremely low dead volumes. The total extracted protein amount using the described protein extraction protocol varies on our tested microorganisms, but the protein amounts extracted from them all show a linear relationship to the amount of biomass used in the test (Figure 1). With two OD*mL (~2 $\times 10^9$ cells) of biomass, we obtained up to 92 μg and 57 ug total proteins from Gram-positive bacteria species of *C. glutamicum* and *S. albus* (Figure 1A), 293 ug and 203 ug total proteins from Gram-negative bacteria species of *E. coli* and *P. putida* (Figure 1B), and 234 ug and 148 ug total proteins from non-filamentous fungi species of *S. cerevisiae* and *R. toruloides* (Figure 1C). The protein amounts are sufficient for typical nano- and standard-flow LC-MS data acquisition methods and can easily be adjusted for applications requiring larger amounts of protein such as deep-proteome analysis by fractionation or post-translational modification characterization experiments. The upper limit on the amount of biomass that can be processed with this protocol is limited by the amount of SDS-alkaline based lysis buffer that can be added to the PCR plate (~25 μL). For applications that require larger amounts of protein, such as multi-dimensional chromatography, the protocol

can easily be adapted to extractions in deep-well plates with correspondingly more lysis buffer and acetone. The protocol can also be scaled down to approximately 0.25 OD*mLs cells for gram negative bacteria and non-filamentous fungi species, and 0.5 OD*mLs cells for Gram-positive bacteria with consistent protein recovery (*n* = 4). Sample types other than microbial cell pellets, such as tissues and complex biofluids, haven't been tested with this protocol and may need additional sample preparation steps. Microbes that are highly resistant to cell lysis may require stronger lysis buffer conditions or increased temperatures. Proteolytic digestion of proteins resulting from these samples, however, are readily suitable for LC-MS analysis without the need of additional desalting steps.

Because of its broad applicability, we tested the protocol on mock mixtures of microbial community samples to assess community biomass composition structure using the metaproteomics analysis method established previously [23]. This method estimates the microbial community structure by calculating the percentage of proteinaceous contribution from each member, which is based on the quantitative measurements of the unique peptides that are identified in each member. High-quality, high-throughput, reproducible metaproteomic data will contribute to wider application of such experiments for microbial community analysis [2,24,25]. The expected microbial community composition results from samples prepared by the protocol is demonstrated in Figures 2 and 3 by using an Agilent 1290 UHPLC coupled to a Thermo Orbitrap Exploris 480 system operating in data-independent acquisition (DIA) mode [21]. From a LC-MS/MS method (15 minute total run time) we identified more than 1100 proteins and 7000 peptides of each microbe from a 12 μg load of the mock microbial community mixture, consistent with previous applications of the digestion protocols [13]. We prepared mixtures of mock microbial communities consisting of two Gram-negative bacteria (*E. coli* and *P. putida*) or two non-filamentous fungi (*S. cerevisiae* and *R. toruloides*) at four biomass ratios. The estimated biomass compositions are reproducible (0.83% standard deviation of the replicates; *n* = 4) and the measured ratios are in close agreement (<3.5%) of the expected ratios as designed (Figure 2, Table 1).

Next, we tested the protocol on a synthetic microbial community of five environmental isolates [22] consisting of one Gram-positive bacterium (ENIGMA isolate: FW305-3-2-15-F-LB2 (GenBank Accession number: OM867407) and four Gram-negative bacteria (ENIGMA isolates: GW456-12-10-14-LB3 (GenBank Accession number: MH795608), GW458-12-9-14-LB2 (GenBank Accession number: MH795601), GW460-LB6 (GenBank Accession number: MH795591), GW460-11-11-14-TSB4 (GenBank Accession number: MH795599)) grown under different experimental conditions to determine composition changes. We started the culture of the five-member microbial community at 20% composition of each and grew them in two culture media, Reasoner's 2A (R2A) [26] and glucose minimal medium.

Table 1

Mix	% measured	% expected		Mix	% measured	% expected
E. coli	4.4 ± 0.2	5		*S. cerevisiae*	8.4 ± 0.1	5
P. putida	95.6 ± 0.2	95		*R. toruloides*	91.6 ± 0.1	95
E. coli	8.2 ± 0.1	10		*S. cerevisiae*	12.1 ± 0.3	10
P. putida	92.8 ± 0.1	90		*R. toruloides*	87.9 ± 0.3	90
E. coli	23.0 ± 0.3	25		*S. cerevisiae*	26.2 ± 0.6	25
P. putida	77.0 ± 0.3	75		*R. toruloides*	73.8 ± 0.6	75
E. coli	48.6 ± 0.5	50		*S. cerevisiae*	52.7 ± 0.8	50
P. putida	51.4 ± 0.5	50		*R. toruloides*	47.3 ± 0.8	50

We observed significant divergence from the initial composition in each culture medium after 24 hours (Figure 3). The GW456-12-10-14-LB3 isolate decreased in both media while GW458-12-9-14-LB2 increased in glucose minimal medium and GW460-LB6 increased significantly in R2A medium. These differences in community structure reflect the impacts of medium compositions on species interactions and dynamics. This protocol is designed for lab-based, culture conditions and synthetic community experiments where complex sample matrices (*e.g.,* feces, soil) are minimized. Additional sample preservation and/or protein extraction methods may be required for environmental samples to minimize protein degradation and maintain sample integrity. Overall, the flexible design of this protocol enables one researcher to prepare

thousands of bottom-up proteomic samples per week. Supporting publications for other monoculture and synthetic community experiments are under development.

Supporting Information

S1: Alkaline-SDS cell lysis with acetone protein precipitation for proteomic sample preparation of microbes in 96-well plate format (PDF). Also available on protocols.io.

Data Availability

The LCMS analysis raw data have been deposited to the ProteomeXchange Consortium data depository at http://www.proteomexchange.org/. They are publicly accessible with the dataset identifier PXD039268.

Acknowledgements

The authors thank Dr. Lauren Lui and Dr. Torben Nielsen for help with the proteome information for the ENIGMA organisms. We also thank Dr. Romy Chakraborty for isolating the ENIGMA organisms and Dr. John-Marc Chandonia for help with the genome information sharing of the ENIGMA organisms.

Funding Statement

The funders had and will not have a role in study design, data collection and analysis, decision to publish, or preparation of the manuscript. The United States Government retains and the publisher, by accepting the article for publication, acknowledges that the United States Government retains a non-exclusive, paid-up, irrevocable, worldwide license to publish or reproduce the published form of this manuscript, or allow others to do so, for United States Government purposes. The views and opinions of the authors expressed herein do not necessarily state or reflect those of the United States Government or any agency thereof. Neither the United States Government nor any agency thereof, nor any of their employees, makes any warranty, expressed or implied, or assumes any legal liability or responsibility for the accuracy,

completeness, or usefulness of any information, apparatus, product, or process disclosed, or represents that its use would not infringe privately owned rights. The proof-of-concept work with mono-cultures and resources were part of the Joint BioEnergy Institute (JBEI; http://www.jbei.org), the extension of the procedure and identification of the sources of error were part of the Agile BioFoundry (ABF; http://agilebiofoundry.org), and the application to synthetic microbial communities was part of Ecosystems and Networks Integrated with Genes and Molecular Assemblies (ENIGMA; http://enigma.lbl.gov), a Science Focus Area led by Lawrence Berkeley National Laboratory. This work was supported by the U.S. Department of Energy, Office of Science, Office of Biological & Environmental Research (JBEI and ENIGMA) and through the Bioenergy Technology Office within the DOE Office of Energy Efficiency and Renewable Energy (ABF) through contract DE-AC02-05CH11231 between Lawrence Berkeley National Laboratory and the U. S. Department of Energy.

Author Information

Corresponding author

*E-mail: cjpetzold@lbl.gov

Author Contributions

Conceptualization: Yan Chen, Christopher J. Petzold.

Investigation: Yan Chen, Jennifer Gin, Stephen Tan, Ying Wang, Markus de Raad.

Supervision: Nathan Hillson, Trent Northen, Paul Adams, Christopher Petzold.

Writing – original draft: Yan Chen, Christopher J. Petzold.

Writing – review & editing: all authors.

Figure Captions

Figure 1. Scatter plots with data points showing the total protein extracted by using the alkaline-acetone sample preparation protocol on different amounts of biomass from: (A) Gram-positive; (B) Gram-negative; and (C) fungal cells. The error bars represent two replicates performed on separate days.

Figure 2. Community proteomic composition analysis of mock mixtures shows accurate estimation of biomass contribution. The stacked bar plots display the percentage of biomass contribution from members in (A) Gram-negative mock community, and (B) Non-filamentous fungi mock community. The error bar shows the standard deviation of calculated composition of each member (n = 4). The LCMS analysis raw data have been deposited to the ProteomeXchange Consortium data depository at http://www.proteomexchange.org/. They are publicly accessible with the dataset identifier PXD039268.

Figure 3. Community proteomic composition analysis shows accurate estimation of biomass contribution of community structure changes upon different culture conditions. The stacked bar plots display the percentage of biomass contribution from members of the synthetic microbial community, and the error bars show the standard deviation of calculated composition of each member (n = 3). Synthetic microbial community shows distinct structure when culturing in different media, Reasoner's 2A (R2A) and glucose minimal medium. The LCMS analysis raw data have been deposited to the ProteomeXchange Consortium data depository at http://www.proteomexchange.org/. They are publicly accessible with the dataset identifier PXD039268.

References

1. Petzold CJ, Chan LJG, Nhan M, Adams PD. Analytics for metabolic engineering. Front Bioeng

Biotechnol. 2015;3: 135. doi:10.3389/fbioe.2015.00135

2. Kleiner M. Metaproteomics: Much More than Measuring Gene Expression in Microbial Communities. mSystems. 2019;4. doi:10.1128/mSystems.00115-19

3. Qian C, Niu B, Jimenez RB, Wang J, Albarghouthi M. Fully automated peptide mapping multi-attribute method by liquid chromatography-mass spectrometry with robotic liquid handling system. J Pharm Biomed Anal. 2021;198: 113988. doi:10.1016/j.jpba.2021.113988

4. Fu Q, Kowalski MP, Mastali M, Parker SJ, Sobhani K, van den Broek I, et al. Highly reproducible automated proteomics sample preparation workflow for quantitative mass spectrometry. J Proteome Res. 2018;17: 420–428. doi:10.1021/acs.jproteome.7b00623

5. Lu X, Wang Z, Gao Y, Chen W, Wang L, Huang P, et al. Autoproteome chip system for fully automated and integrated proteomics sample preparation and peptide fractionation. Anal Chem. 2020. doi:10.1021/acs.analchem.0c00752

6. Lee J, Kim H, Sohn A, Yeo I, Kim Y. Cost-Effective Automated Preparation of Serum Samples for Reproducible Quantitative Clinical Proteomics. J Proteome Res. 2019;18: 2337–2345. doi:10.1021/acs.jproteome.9b00023

7. Dayon L, Núñez Galindo A, Corthésy J, Cominetti O, Kussmann M. Comprehensive and Scalable Highly Automated MS-Based Proteomic Workflow for Clinical Biomarker Discovery in Human Plasma. J Proteome Res. 2014;13: 3837–3845. doi:10.1021/pr500635f

8. Arul A-B, Byambadorj M, Han N-Y, Park JM, Lee H. Development of an Automated, High-throughput Sample Preparation Protocol for Proteomics Analysis. Bull Korean Chem Soc. 2015;36: 1791–1798. doi:10.1002/bkcs.10338

9. Müller T, Kalxdorf M, Longuespée R, Kazdal DN, Stenzinger A, Krijgsveld J. Automated sample preparation with SP3 for low-input clinical proteomics. Mol Syst Biol. 2020;16: e9111. doi:10.15252/msb.20199111

10. Liang Y, Acor H, McCown MA, Nwosu AJ, Boekweg H, Axtell NB, et al. Fully Automated Sample Processing and Analysis Workflow for Low-Input Proteome Profiling. Anal Chem. 2020. doi:10.1021/acs.analchem.0c04240

11. Burns AP, Zhang Y-Q, Xu T, Wei Z, Yao Q, Fang Y, et al. A Universal and High-Throughput Proteomics Sample Preparation Platform. Anal Chem. 2021. doi:10.1021/acs.analchem.1c00265

12. Chen Y, Guenther JM, Gin JW, Chan LJG, Costello Z, Ogorzalek TL, et al. Automated “Cells-To-Peptides” Sample Preparation Workflow for High-Throughput, Quantitative Proteomic Assays of Microbes. J Proteome Res. 2019;18: 3752–3761. doi:10.1021/acs.jproteome.9b00455

13. Chen Y, Kaplan Lease N, Gin J, Ogorzalek T, Adams P, Hillson N, et al. Modular automated bottom-up proteomic sample preparation for high-throughput applications. PLoS ONE. 2022;17: e0264467.

14. Doellinger J, Schneider A, Hoeller M, Lasch P. Sample Preparation by Easy Extraction and Digestion (SPEED) - A Universal, Rapid, and Detergent-free Protocol for Proteomics Based on Acid Extraction. Mol Cell Proteomics. 2020;19: 209–222. doi:10.1074/mcp.TIR119.001616

15. Sandbaumhüter FA, Nezhyva M, Eriksson O, Engberg A, Kreuger J, Andrén PE, et al. Well-Plate

μFASP for Proteomic Analysis of Single Pancreatic Islets. J Proteome Res. 2022. doi:10.1021/acs.jproteome.2c00047

16. Loroch S, Kopczynski D, Schneider AC, Schumbrutzki C, Feldmann I, Panagiotidis E, et al. Toward Zero Variance in Proteomics Sample Preparation: Positive-Pressure FASP in 96-Well Format (PF96) Enables Highly Reproducible, Time- and Cost-Efficient Analysis of Sample Cohorts. J Proteome Res. 2022. doi:10.1021/acs.jproteome.1c00706

17. Nickerson JL, Doucette AA. Rapid and quantitative protein precipitation for proteome analysis by mass spectrometry. J Proteome Res. 2020;19: 2035–2042. doi:10.1021/acs.jproteome.9b00867

18. Doucette A, Crowell A. Precipitation of Detergent-Containing Samples for Top-Down and Bottom-Up Proteomics. In: Y. Abdurakhmonov I, editor. Proteomics technologies and applications. IntechOpen; 2019. doi:10.5772/intechopen.85547

19. Crowell AMJ, Wall MJ, Doucette AA. Maximizing recovery of water-soluble proteins through acetone precipitation. Anal Chim Acta. 2013;796: 48–54. doi:10.1016/j.aca.2013.08.005

20. Chen Y, Ogorzalek T, Kaplan Lease N, Gin J, J Petzold C. Automated Protein Normalization and Tryptic Digestion on a Biomek-NX Liquid Handler System v2. 2022. doi:10.17504/protocols.io.b3gtqjwn

21. Chen Y. Discovery proteomic (DIA) LC-MS/MS data acquisition and analysis v2. 2022. doi:10.17504/protocols.io.e6nvwk1z7vmk/v2

22. Saichek NR, Wang Y, Kosina SM, Bowen BP, Chakraborty R, Northen TR. Conservation of isolate substrate preferences in mixed communities revealed through ribosomal marker protein profiling. BioRxiv. 2021. doi:10.1101/2021.05.12.443848

23. Kleiner M, Thorson E, Sharp CE, Dong X, Liu D, Li C, et al. Assessing species biomass contributions in microbial communities via metaproteomics. Nat Commun. 2017;8: 1558. doi:10.1038/s41467-017-01544-x

24. Salvato F, Hettich RL, Kleiner M. Five key aspects of metaproteomics as a tool to understand functional interactions in host-associated microbiomes. PLoS Pathog. 2021;17: e1009245. doi:10.1371/journal.ppat.1009245

25. Van Den Bossche T, Kunath BJ, Schallert K, Schäpe SS, Abraham PE, Armengaud J, et al. Critical Assessment of MetaProteome Investigation (CAMPI): a multi-laboratory comparison of established workflows. Nat Commun. 2021;12: 7305. doi:10.1038/s41467-021-27542-8

26. Reasoner DJ, Geldreich EE. A new medium for the enumeration and subculture of bacteria from potable water. Appl Environ Microbiol. 1985;49: 1–7. doi:10.1128/aem.49.1.1-7.1985

Figure 1

Click here to access/download;Figure;Fig1-Alkaline-SDS_cell_lysis_protocol.eps

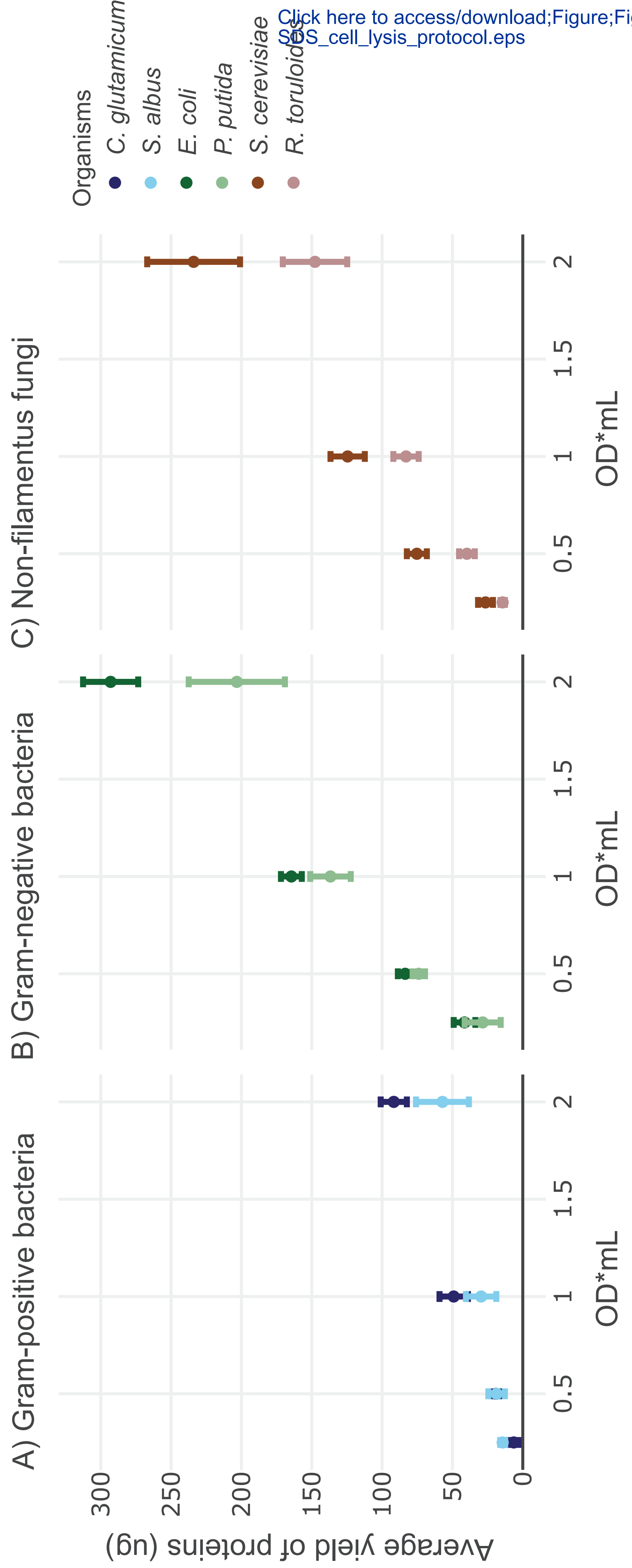

Figure 1

Figure 2

Click here to access/download;Figure;Fig2-Alkaline-SDS_cell_lysis_protocol.eps

Figure 2

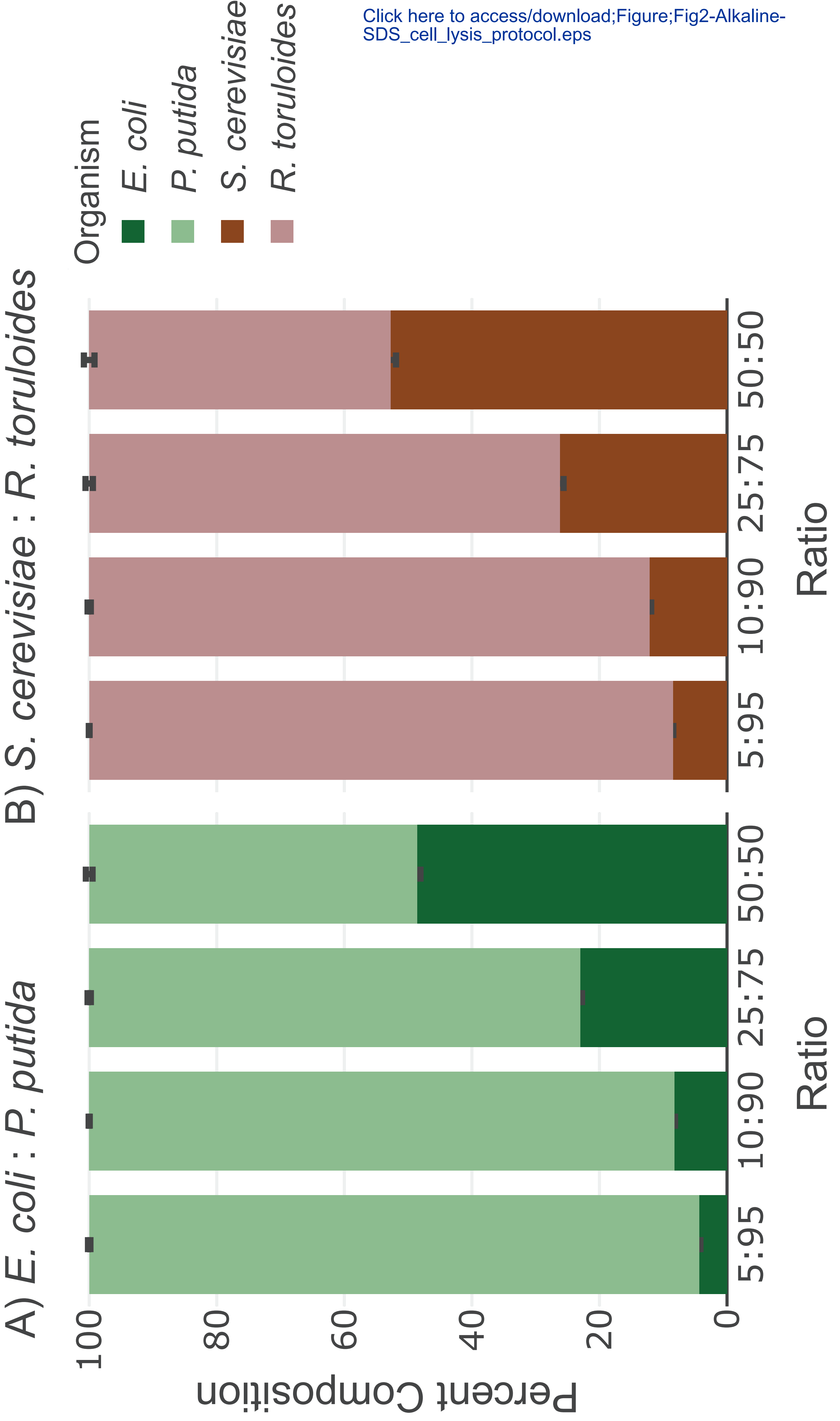

Click here to access/download;Figure;Fig3-Alkaline-SDS_cell_lysis_protocol.eps

Figure 3

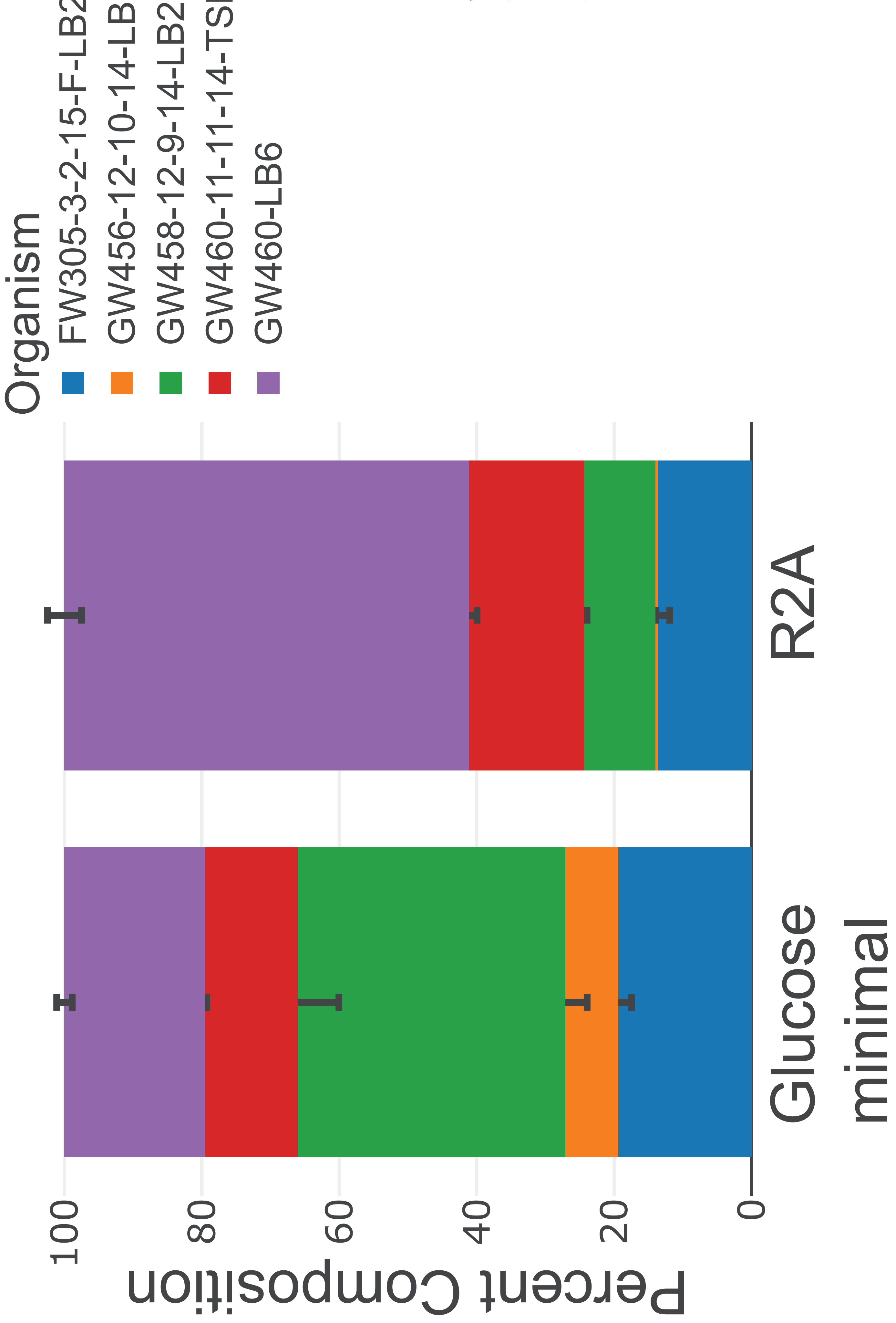

Click here to access/download
Supporting Information
S1_alkaline-sds-cell-lysis-of-microbes-with-acetone.pdf
